# A transformer-based reinforcement learning framework for autonomous training of industrial robotic manipulators

**DOI:** 10.3389/frai.2026.1922657

**Published:** 2026-09-24

**Authors:** Balnur Kenjayeva, Alibek Galimov

**Affiliations:** 1 International University of Tourism and Hospitality, Turkistan, Kazakhstan; 2 Joldasbekov Institute of Mechanics and Engineering, Almaty, Kazakhstan

**Keywords:** deep learning, intelligent manufacturing, reinforcement learning, robotic manipulators, trajectory optimization, transformers

## Abstract

**Introduction:**

Autonomous robotic manipulation in modern manufacturing requires control policies that simultaneously achieve precision, adaptation, operational safety, and multi-task capability.

**Methods:**

This paper proposes a Transformer-Based Reinforcement Learning Network (TRL-Net) that combines temporal state encoding, task-conditioned representation, actor-critic reinforcement learning, domain-randomized simulation, and safety-constrained action projection. The evaluation is limited to ten simulated industrial manipulation tasks.

**Results:**

The reported point estimates are a 98.6% task success rate, 0.81 mm average positioning error, 0.0% collision rate in the evaluated simulation episodes, 0.42 m/s³ trajectory jerk, and 4.2 ms policy inference time.

**Discussion:**

These findings are consistent with improved temporal control and multi-task performance relative to the reported baselines. However, the archived experimental record does not contain real-robot trials, component-wise ablations, seed-level dispersion, complete hyperparameters, or hardware specifications. The conclusions are therefore restricted to the reported simulation setting, and the safety layer is interpreted as enforcing configured simulator constraints rather than guaranteeing safe operation on physical industrial hardware.

## 1 Introduction

The rapid advancement of Industry 4.0 has significantly accelerated the deployment of industrial robotic manipulators in modern manufacturing environments. Robots are now extensively utilized in applications such as material handling, assembly, welding, polishing, palletizing, machine tending, and precision inspection, where high productivity, repeatability, and operational flexibility are essential. Despite these advances, most industrial manipulators continue to rely on manually programmed trajectories or teach-pendant programming, requiring considerable engineering effort whenever production requirements or workspace configurations change. Such conventional programming approaches are often time-consuming, task-specific, and incapable of adapting autonomously to dynamic industrial environments. Consequently, developing intelligent robotic systems capable of learning complex manipulation skills directly through interaction has become an important research topic in industrial automation and intelligent manufacturing (Hu et al., 2024).

Traditional robot motion planning algorithms, including linear interpolation, polynomial trajectory generation, spline-based methods, and optimization techniques, have demonstrated satisfactory performance in structured environments. Sampling-based planners such as Rapidly-exploring Random Trees (RRT*) and optimization-based methods including Covariant Hamiltonian Optimization for Motion Planning (CHOMP) and Stochastic Trajectory Optimization for Motion Planning (STOMP) further improve trajectory quality while satisfying kinematic constraints (Yuan et al., 2024). Nevertheless, these approaches generally depend on accurate mathematical models, handcrafted cost functions, and manually tuned parameters, which restrict their ability to generalize across multiple industrial tasks or adapt to environmental uncertainties. Furthermore, conventional planners optimize predefined trajectories rather than enabling robots to acquire reusable manipulation skills through experience (Fazlollahtabar, 2025).

Deep reinforcement learning (DRL) has recently emerged as an effective paradigm for autonomous robotic learning by allowing agents to optimize control policies through continuous interaction with the environment. Representative algorithms such as Proximal Policy Optimization (PPO), Deep Deterministic Policy Gradient (DDPG), Soft Actor-Critic (SAC), and Twin Delayed Deep Deterministic Policy Gradient (TD3) have achieved promising results in robotic manipulation and continuous control tasks (Elseiagy et al., 2026). However, most existing DRL algorithms make decisions primarily from the current observation, limiting their ability to capture long-term temporal dependencies during complex sequential manipulation. Consequently, these methods often exhibit slow convergence, unstable training, limited sample efficiency, and reduced generalization when multiple industrial operations must be learned within a unified policy (Momynkulov et al., 2025).

Transformer-based decision methods span several distinct learning settings. Decision Transformer learns an offline return-conditioned autoregressive policy from fixed trajectories, whereas Trajectory Transformer learns a trajectory distribution and uses beam search for offline planning (Chen et al., 2021; Janner et al., 2021). Robot Transformers such as RT-1 and PerAct instead learn multi-task visuomotor policies primarily from demonstrations; their principal contributions concern data scaling, language conditioning, and spatial action representation rather than online actor-critic optimization with a constraint-projection layer (Brohan et al., 2023; Shridhar et al., 2023). These differences are important because offline sequence modeling, imitation learning, and online reinforcement learning impose different data, safety, and deployment assumptions.

TRL-Net is positioned as an online, continuous-control actor-critic architecture rather than as a Decision Transformer or a demonstration-trained Robot Transformer. Its contribution is the integrated interaction of four modules: (1) temporal self-attention encodes a window of proprioceptive, pose, force, gripper, and progress observations; (2) a learned task token conditions one shared encoder for ten manufacturing operations; (3) actor and value heads optimize a continuous control policy; and (4) an external projection step modifies commands that violate configured joint, velocity, torque, clearance, or workspace constraints. Domain randomization broadens the simulated training distribution. The novelty claim is therefore limited to this systems-level integration and evaluation, not to a new self-attention operator, a new actor-critic theorem, or a demonstrated sim-to-real transfer method.

## 2 Related works

Industrial robotic manipulation has traditionally relied on model-based motion planning algorithms that generate feasible trajectories while satisfying kinematic and dynamic constraints (Li et al., 2025). Classical approaches such as linear interpolation, cubic spline, quintic polynomial, and B-spline trajectory generation remain widely adopted because of their computational efficiency and smooth trajectory characteristics (Qu et al., 2026; Kumar et al., 2026). More advanced planning methods, including sampling-based algorithms such as Rapidly-exploring Random Trees (RRT*) and optimization-based techniques including Covariant Hamiltonian Optimization for Motion Planning (CHOMP) and Stochastic Trajectory Optimization for Motion Planning (STOMP), have further improved motion quality through obstacle avoidance and cost optimization (Zhou and Zhang, 2025). Although these methods provide reliable trajectory generation in structured environments, they typically require accurate environment models, manually designed objective functions, and extensive parameter tuning (Kaigom, 2023). Furthermore, they optimize predefined trajectories rather than enabling robots to autonomously acquire manipulation skills from experience, limiting their adaptability to dynamic industrial scenarios.

Deep reinforcement learning (DRL) has emerged as a powerful alternative for autonomous robot control by allowing manipulators to learn optimal control policies through trial-and-error interactions with the environment. Value-based and policy-gradient algorithms have demonstrated remarkable success in continuous robotic manipulation tasks, particularly Proximal Policy Optimization (PPO), Deep Deterministic Policy Gradient (DDPG), Soft Actor-Critic (SAC), and Twin Delayed Deep Deterministic Policy Gradient (TD3) (Giral et al., 2026; Bilal et al., 2024). PPO improves policy stability through clipped objective optimization, whereas DDPG extends deterministic policy gradients to continuous action spaces (Krohn et al., 2026; Huang et al., 2023; Ray, 2026). SAC introduces entropy maximization to enhance exploration and learning robustness (Zhao et al., 2024), while TD3 reduces value overestimation through delayed policy updates and double critics, resulting in improved stability for continuous control problems (Yao Z. et al., 2025; Yao T. et al., 2025). Despite these advances, conventional DRL algorithms generally operate using only the current observation or a limited history of states, making it difficult to capture long-term temporal dependencies required for complex industrial manipulation involving multiple sequential actions.

Decision Transformer conditions a causal sequence model on desired return, states, and past actions without fitting a conventional actor or critic, while Trajectory Transformer models joint sequences of states, actions, and rewards and performs beam-search planning (Chen et al., 2021; Janner et al., 2021). RT-1 maps short image histories and language instructions to discretized robot actions using large demonstration datasets, and PerAct predicts discretized 6-DoF actions from language-conditioned 3D voxel representations through behavior cloning (Brohan et al., 2023; Shridhar et al., 2023). DreamerV3 represents a complementary model-based family that learns a world model and improves behavior through imagined trajectories rather than through a Transformer safety projection (Hafner et al., 2023). None of these distinctions alone establishes superiority; they define different training regimes and motivate the technical positioning summarized in Table 1.

**Table 1:** Technical positioning of representative transformer, model-based, and safe robotic learning approaches.

Approach	Learning and conditioning mechanism	Safety mechanism	Difference from or limitation relative to TRL-Net
Decision transformer	Offline return-conditioned causal sequence modeling from fixed trajectories.	No task-specific action projection in the original method.	No online actor-critic optimization; not designed around industrial robot constraints.
Trajectory transformer	Offline trajectory distribution modeling with beam-search planning.	Safety may enter through planning objectives but is not an integrated projection layer.	Model-based offline planner rather than a shared task-conditioned continuous actor-critic policy.
RT-1	Language-conditioned Transformer policy trained from large multi-task robot demonstrations.	Relies on data and execution infrastructure; no formal feasibility projection in the core policy.	Demonstration-driven and perception-centered; requires substantial real-robot data.
PerAct	Language-conditioned 3D voxel Transformer trained by behavior cloning.	Motion execution can use an external planner; no online safe-RL update.	Discrete 6-DoF imitation policy rather than temporal online actor-critic learning.
DreamerV3	Model-based RL with a learned world model and imagined rollouts.	No industrial action-projection module in the general algorithm.	Different model-based mechanism; a matched-budget comparison was not available.
Recovery RL	Task policy plus learned recovery policy for constraint-violating regions.	Recovery intervention based on a learned safety critic/policy.	Safe RL comparator without the temporal task-conditioned Transformer integration.
TRL-Net	Temporal observation Transformer, learned task token, and continuous actor/value heads.	One-step feasibility projection over configured robot constraints.	Evaluated only in simulation; no hardware validation, complete ablation, or seed-level uncertainty.

Prior work leaves an integration gap at the intersection of online temporal reinforcement learning, task-conditioned industrial control, and deterministic action feasibility checks. Safe RL methods such as Recovery RL can intervene through a separately learned recovery policy, but they do not combine a shared temporal Transformer and task token for the industrial task set studied here (Thananjeyan et al., 2021). Conversely, multi-task Robot Transformers provide strong semantic or spatial conditioning but usually depend on curated demonstrations and do not expose an actor-critic update coupled to explicit joint, torque, collision-clearance, and workspace projection. TRL-Net addresses this integration gap in simulation, while real-hardware safety, statistical reliability, and component-level causal evidence remain unresolved.

The individual role of each TRL-Net module is as follows. Temporal encoding supplies motion and contact history; task conditioning selects task-relevant behavior without creating ten separate encoders; the actor-critic heads provide the reinforcement-learning objective; domain randomization perturbs the training distribution; and safety correction separates command feasibility from reward shaping. Because the supplied record contains no component-wise ablation, the present results support the performance of the complete pipeline but do not isolate the causal contribution of any one module.

Recent hydraulic-control studies provide a useful complementary comparison. Model-based reinforcement learning for electrohydraulic position servos combines actor-critic adaptation with a robust control structure and system-level stability analysis; data-driven control of a 6-DoF hydraulic manipulator learns tracking actions along task trajectories; and model-data hybrid control combines available hydraulic dynamics with learned compensation for difficult mechanical dynamics (Yao et al., 2023; Yao et al., 2024; Yao Z. et al., 2025). These studies emphasize tracking guarantees and physical hydraulic experiments. TRL-Net instead emphasizes temporal multi-task representation and command projection, but does not presently provide comparable Lyapunov stability guarantees or hardware evidence.

Transformer-based robotic control has been investigated for stable grasping (Puang et al., 2024). Related research combines Transformer representations with imitative reinforcement learning for multirobot path planning (Chen et al., 2023). Multi-agent reinforcement learning has also been studied for coordinating multiple machine-tending operations (Abdalwhab et al., 2026). Software-defined self-learning control provides another industrial-robot application of reinforcement learning (Moon et al., 2026).

Further applications include object recognition and obstacle avoidance for multiple mobile robots using Swin Transformers and deep reinforcement learning (Fu et al., 2025). Transformer-based planning has been examined for single-arm and dual-arm robots in dynamic environments (Wang et al., 2025). Multimodal learning has also been investigated for autonomous pallet handling in dynamic warehouse settings (Zhao et al., 2025). Energy-sensitive robotic manipulation of articulated components offers an additional application context (Tao et al., 2026).

Adjacent research includes reinforcement learning for decision-making at unsignalized intersections (Al-Sharman et al., 2025). Broader machine-learning approaches have been reviewed for autonomous vehicle navigation (Shrivastava et al., 2026). Other application domains include hybrid reinforcement learning for health-related decision-making (Abdullah et al., 2026). Vision Transformers have also been applied to robotic surgical skill assessment (Zhang et al., 2024). Attention mechanisms have been explored in reinforcement learning for humanoid locomotion (Chu et al., 2025). These application contexts differ from the industrial manipulation evaluation considered here and are not treated as direct performance baselines.

For transfer beyond simulation, visual domain randomization provides a related approach to varying simulated observations (Tobin et al., 2017). Dynamics randomization addresses variation in the physical parameters underlying learned robotic control (Peng et al., 2018). These studies motivate consideration of simulation variability but do not establish hardware transfer for the present framework.

## 3 Materials and methods

The Materials and Methods section describes the proposed Transformer-Based Reinforcement Learning framework for autonomous industrial robotic manipulation. It presents the overall learning architecture, the Transformer Reinforcement Learning Network (TRL-Net), multi-task industrial training environment, safety-constrained action correction mechanism, and experimental setup. The section further introduces the mathematical formulation of the reinforcement learning problem, temporal Transformer encoding, policy optimization, reward design, and safety constraints, providing the theoretical and implementation details required to reproduce the proposed framework.

### 3.1 Overall framework

The proposed Transformer-Based Reinforcement Learning Framework is designed to enable autonomous training of industrial robotic manipulators capable of performing diverse manipulation tasks while satisfying strict safety constraints. Unlike conventional reinforcement learning methods that rely only on the current system state, the proposed framework incorporates temporal state representations through a Transformer encoder, allowing the policy to exploit long-range dependencies in robotic motion sequences. The overall architecture of the proposed system is illustrated in Figure 1, where the complete learning pipeline consists of task specification, simulation environment, state observation, Transformer Reinforcement Learning Policy (TRL-Net), action generation, safety verification, robotic execution, experience replay, policy optimization, and reward evaluation.

**Figure 1:**
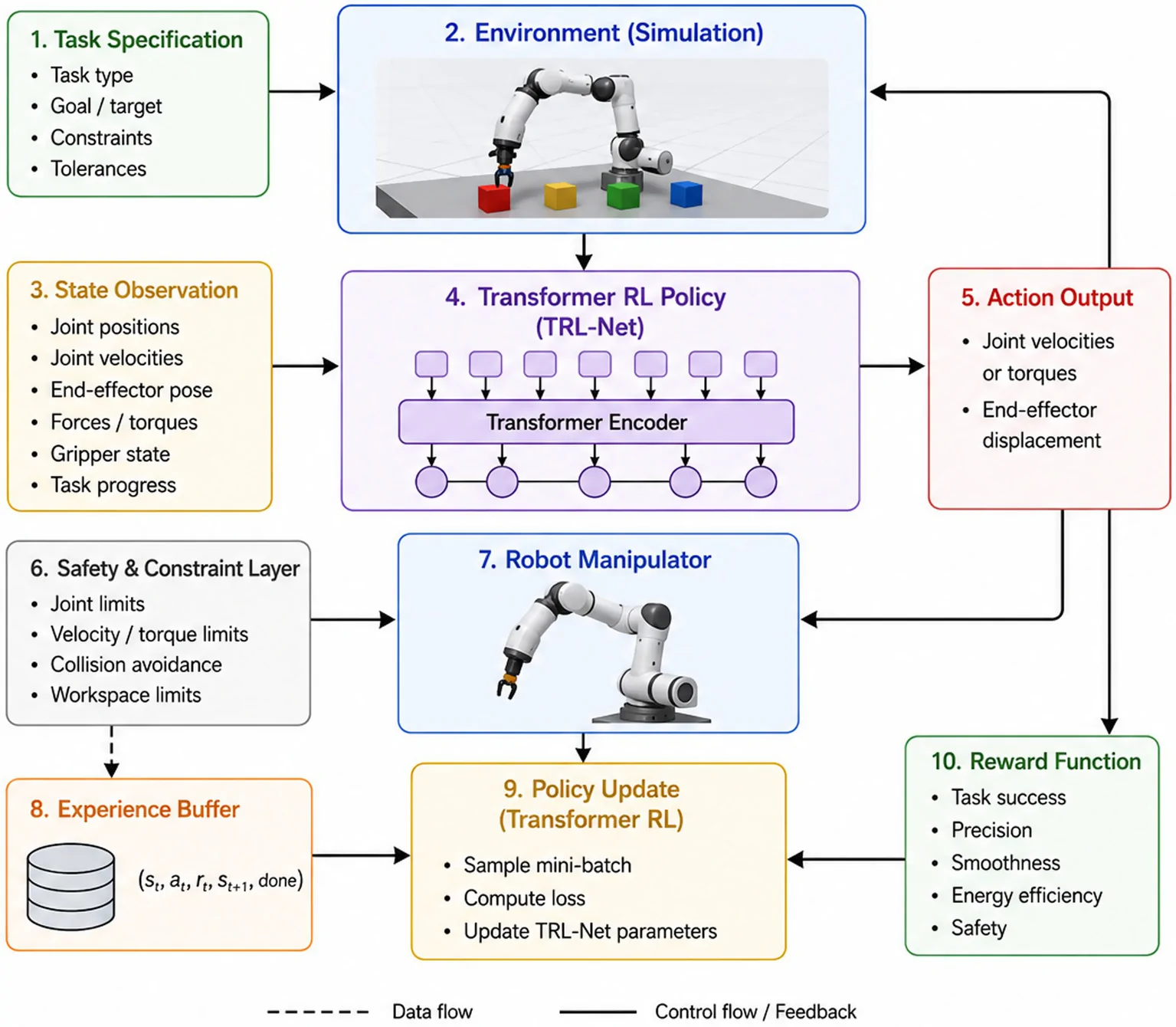
Overall framework of the proposed transformer-based robotic training system.

The training process begins with task specification, where the desired industrial operation is defined according to the manipulation objective. Each task is represented by Equation 1:

τ={g,C,ε} (1)

Where g denotes the target objective, C represents task-specific constraints, and ϵ defines the allowable execution tolerance.

The proposed framework supports multiple industrial applications including pick-and-place, assembly, screw driving, spot welding, arc welding, polishing, machine tending, bin picking, palletizing, and multi-stage assembly, as illustrated in Figure 2. During training, all tasks are executed inside a high-fidelity physics simulation that accurately models robot dynamics, contact interactions, actuator characteristics, and environmental disturbances.

**Figure 2:**
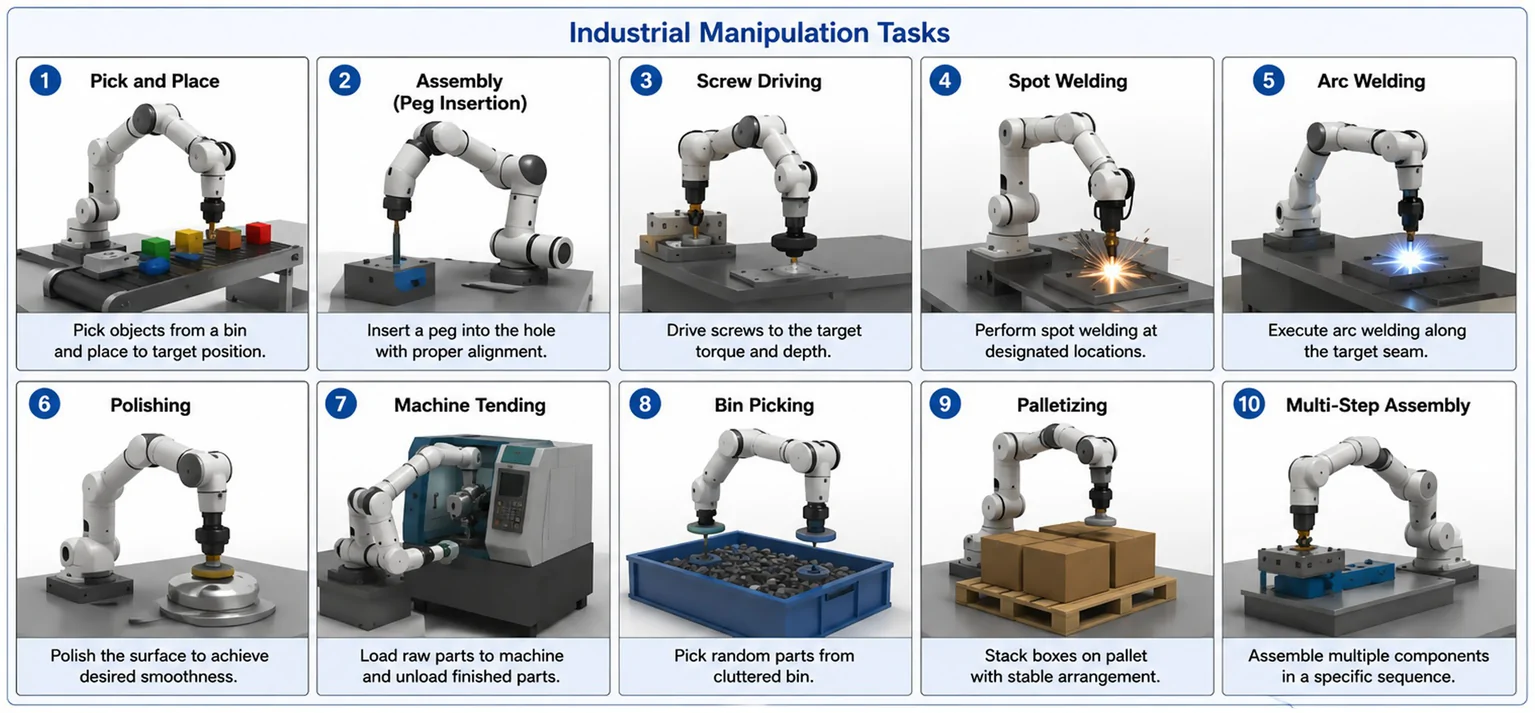
Multi-task industrial training environment.

To improve policy generalization, extensive domain randomization is incorporated throughout the simulation process. For every training episode, lighting conditions, camera viewpoints, object geometry, textures, masses, friction coefficients, actuator delays, and robot dynamics are randomly sampled. Let $\omega =\{\omega_{l},\omega_{c},\omega_{o},\omega_{p},\omega_{r}\}$ represent the randomized environment parameters corresponding to illumination, camera settings, object properties, physics parameters, and robot dynamics, respectively. The simulated environment can therefore be expressed by Equation 2:

E(ω) (2)

Which generates diverse training conditions that reduce the sim-to-real performance gap.

At every control cycle, the simulator provides a multidimensional observation vector containing robot joint positions, joint velocities, end-effector pose, measured forces, gripper status, and task progress. The complete state vector is represented as

$$\begin{array}{cccc} & s_{t}=[q_{t},{\dot{q}}_{t},x_{t},f_{t},g_{t},p_{t}] & & \end{array}$$ (3)

Where $q_{t}$ and ${\dot{q}}_{t}$ denote joint positions and velocities, $x_{t}$ is the Cartesian end-effector pose, $f_{t}$ represents measured force/torque information, $g_{t}$ is the gripper state, and $p_{t}$ denotes task completion progress.

Rather than processing only the current observation, the proposed method constructs he temporal history defined in Equation 4:


$$S_{t}=\{s_{t-T+1},\ldots ,s_{t}\}$$ (4)

Where T denotes the sequence length. This sequence preserves motion history and enables the Transformer policy to model temporal dependencies across multiple decision steps.

The observation sequence together with the task embedding is forwarded to the Transformer Reinforcement Learning Policy (TRL-Net) shown in Figure 3, which predicts continuous robot actions according to Equation 5:

$$\begin{array}{cccc} & a_{t}=\pi_{\theta }(S_{t},\tau ) & & \end{array}$$ (5)

Where $\pi_{\theta }(\cdot )$ represents the parameterized Transformer policy.

**Figure 3:**
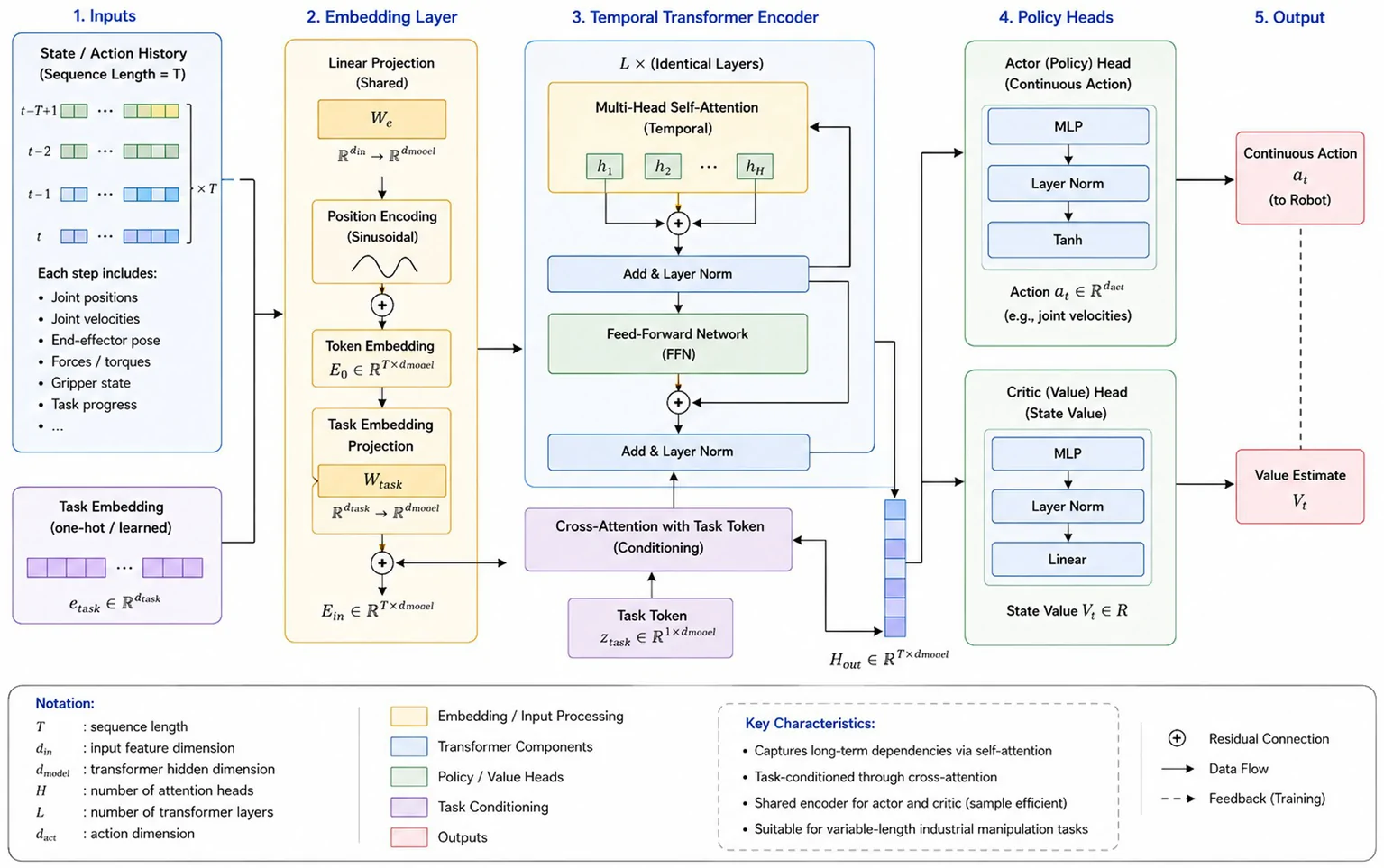
Architecture of the Transformer Reinforcement Learning Policy (TRL-Net).

Since reinforcement learning policies may occasionally generate unsafe commands during exploration, every predicted action is processed by the safety layer illustrated in Figure 4 before execution. The safety module verifies joint limits, velocity constraints, torque limits, collision avoidance, and workspace boundaries. When constraint violations occur, the action is projected onto the nearest feasible solution before being transmitted to the robot.

**Figure 4:**
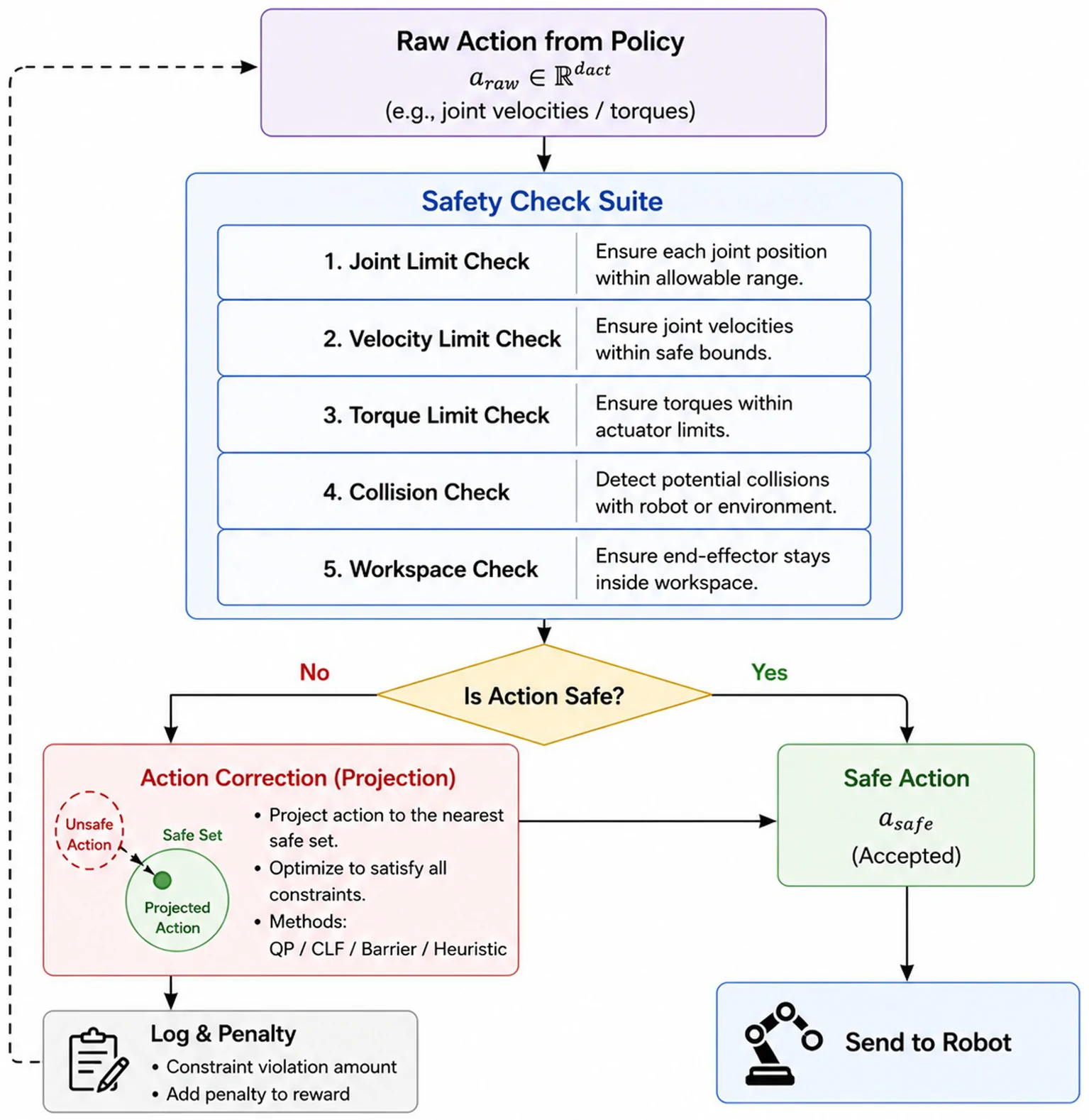
Safety-constrained action correction module.

After execution, the simulator returns the next observation and reward. The transition $\left(s_{t},a_{t},r_{t},s_{t+1}\right)$ is stored inside the experience replay buffer, enabling mini-batch optimization during policy learning. The reward combines task completion, positioning accuracy, motion smoothness, energy efficiency, and safety performance. Consequently, the proposed framework simultaneously learns efficient, accurate, and safe robotic behaviors suitable for industrial manipulation.

### 3.2 Transformer reinforcement learning policy (TRL-Net)

The principal methodological contribution is the coupling of a shared temporal representation with task conditioning, actor-value learning, and an external feasibility projection. The Transformer block itself follows standard multi-head self-attention. The task token changes the encoded representation used by both policy heads, whereas the safety module acts after the raw policy output and can therefore correct a command even when the reward model has not discouraged that command strongly enough.

The network consists of five major components:

1. Input representation
2. Embedding layer
3. Temporal Transformer encoder
4. Actor-Critic policy heads
5. Continuous action generation

#### 3.2.1 Input representation

Each observation sequence consists of the previous T robot states as defined in Equation 6:

$$X=[s_{t-T+1},\ldots ,s_{t}]\in {\mathbb{R}}^{T\times d_{\text{in}}}$$ (6)

Where $d_{\text{in}}$ denotes the input feature dimension.

Each state contains

- joint positions
- joint velocities
- end-effector pose
- force/torque measurements
- gripper state
- task progress

Unlike conventional reinforcement learning algorithms that only observe $s_{t}$ , the proposed method processes the complete temporal sequence, enabling the Transformer to infer motion trends, dynamic behaviors, and manipulation intentions.

#### 3.2.2 State history encoding

The raw observations are first projected into the Transformer latent space through a shared linear embedding in Equation 7:

$$\begin{array}{cccc} & E=X{W_{e}}^{T}+b_{e} & & \end{array}$$ (7)

Where

$$W_{e}\in {\mathbb{R}}^{d_{\text{model}}\times d_{\text{in}}}$$

To preserve temporal ordering, sinusoidal positional encoding is added according to Equation 8:

$$E_{p}=E+\mathrm{PE}$$ (8)

Where

$$\mathrm{PE}(\mathrm{pos},2i)=\sin(\frac{\mathrm{pos}}{10000^{2i/d_{\text{model}}}})$$

$$\mathrm{PE}(\mathrm{pos},2i+1)=\cos(\frac{\mathrm{pos}}{10000^{\frac{2i}{d_{\text{model}}}}})$$

The resulting sequence maintains chronological information while remaining invariant to sequence length (Govinda et al., 2025; Ma et al., 2025).

#### 3.2.3 Task embedding

Industrial robots frequently switch among multiple tasks requiring different objectives and constraints. Therefore, the proposed framework introduces a dedicated task embedding illustrated in Figure 3.

The task embedding is projected into the Transformer feature space according to Equation 9:

$$z_{\text{task}}=W_{\text{task}}e_{\text{task}}$$ (9)

The task token conditions the Transformer encoder through cross-attention, enabling a shared policy to adapt its behavior according to the assigned industrial task without retraining separate policies.

#### 3.2.4 Temporal transformer encoder

The embedded observation sequence is processed by L stacked Transformer encoder layers. Each layer consists of multi-head temporal self-attention followed by a feed-forward network and residual normalization.

For each attention head,

$$Q=XW_{Q},K=XW_{K},V=XW_{V}$$

Scaled dot-product attention is computed using Equation 10:

$$\begin{array}{cccc} & \text{Attention}(Q,K,V)=\text{Softmax}(\frac{QK^{T}}{\sqrt{d_{k}}})V & & \end{array}$$ (10)

Outputs from multiple attention heads are concatenated according to Equation 11:

$$\begin{array}{cccc} & H=\text{Concat}(h_{1},\ldots ,h_{H})W_{O} & & \end{array}$$ (11)

Residual connections and feed-forward transformations produce the latent representation in Equation 12:

$$\begin{array}{cccc} & H_{\text{out}}=\text{Transformer}(X,z_{\text{task}}) & & \end{array}$$ (12)

Which captures temporal motion patterns, contact events, force evolution, and task progress throughout the manipulation sequence.

#### 3.2.5 Actor-critic policy heads

The encoded feature representation is shared between the actor and critic networks.

The actor predicts continuous robot actions according to Equation 13:

$$\begin{array}{cccc} & a_{t}=\pi_{\theta }(H_{\text{out}}) & & \end{array}$$ (13)

Where the output consists of joint velocities or joint torques depending on the selected controller.

The critic estimates the expected cumulative return using Equation 14:

$$\begin{array}{cccc} & V_{t}=V_{\phi }(H_{\text{out}}) & & \end{array}$$ (14)

Providing the value estimate required for policy optimization.

Sharing the Transformer encoder significantly reduces computational complexity while improving sample efficiency because both policy and value estimation exploit the same temporal features.

### 3.3 Implementation details and reproducibility boundary

Equations 3, 4, 6 define the observation history symbolically. For an n-joint robot, the observation contains joint position and velocity vectors, Cartesian end-effector pose, a measured force/torque vector, gripper state, and normalized task progress. Equation 13 produces an n-dimensional continuous joint command that is bounded by the policy output activation and rescaled by the selected controller limits. The archived record does not identify whether the reported experiments used joint velocity or joint torque as the final command; both modes appear in the original description. This ambiguity must be resolved from the source code before independent reproduction.

The conceptual actor update follows the policy-gradient direction $\eta_{\pi }\;\hat{A}\;\nabla_{\theta }\log\pi_{\theta }(a_{t}|H_{\text{out}},\tau )$ , while the critic parameters *φ* minimize $\frac{1}{2}{\left[V_{\phi }(H_{\text{out}},\tau )\hat{R}\right]}^{2}$ , where Â is an advantage estimate and R˄ is a return target. Equations 13, 14, however, specify only the policy and value outputs. The exact loss variant, return estimator, optimizer, learning rates, discount factor, entropy or regularization terms, replay correction, and update schedule were not recorded in the manuscript supplied for revision.

The source manuscript also does not report numerical values for sequence length, embedding dimension, attention heads, Transformer depth, feed-forward width, dropout, batch and replay sizes, reward weights, penalty coefficient, control frequency, robot and simulator versions, domain-randomization intervals, training hardware, parameter count, memory use, wall-clock training time, or random seeds. The available computational quantities are 0.58 million episodes to convergence and 4.2 ms inference time; without hardware specifications these values are not portable performance guarantees. These omissions are explicitly disclosed rather than being filled with reconstructed or assumed values.

### 3.4 Multi-task industrial training environment

The robotic learning environment illustrated in Figure 2 contains ten representative industrial manipulation tasks covering assembly, material handling, welding, machining, polishing, and logistics operations. Each task is initialized using randomized environmental conditions to increase policy robustness.

During each episode, the randomization vector includes visual factors (illumination, camera pose, textures, and background), object factors (geometry, mass, and initial pose), contact factors (friction, restitution, stiffness, and damping), and actuator factors (joint friction, command delay, gain, and payload-dependent dynamics). A fair implementation samples the same parameter vector for all methods within a matched evaluation episode. Numerical lower and upper bounds were not retained in the supplied experimental record, so the robustness envelope and reproducibility of the reported domain randomization cannot be independently established from this manuscript alone.

The robot interacts with the environment until one of the following termination conditions is satisfied:

- successful task completion
- collision
- workspace violation
- timeout

The reward function combines multiple performance objectives according to Equation 15:


$$\begin{array}{cccc} & \begin{array}{l} r_{t}=\alpha R_{\text{task}}+\beta R_{\text{precision}}+\gamma R_{\text{smooth}}\\ +\delta R_{\text{energy}}+\eta R_{\text{safety}} \end{array} & & \end{array}$$ (15)

Where the weighting coefficients satisfy α+β+γ+δ+η=1 . This formulation encourages accurate task completion while minimizing excessive motion, energy consumption, and unsafe behaviors.

### 3.5 Safety-constrained action correction

Although reinforcement learning improves exploration, unrestricted exploration may generate unsafe robot commands. Therefore, every predicted action is processed through the safety-constrained correction framework illustrated in Figure 4.

The generated action $a_{\mathrm{raw}}$ is first evaluated against five constraint categories:

- joint limits
- velocity limits
- torque limits
- collision avoidance
- workspace boundaries

The feasible action set is defined in Equation 16:

$$\begin{array}{cccc} & A_{\text{safe}}=\{a|g_{i}(a)\leq 0,i=1,\ldots ,m\} & & \end{array}$$ (16)

Where each constraint function $g_{i}(\cdot )$ represents one safety requirement.

For a control interval Δt and candidate joint command a, the configured feasible set requires $q_{\min}\leq q_{t}+\mathrm{\Delta t}\;a\leq q_{\max}$ , $|a_{j}|\leq v_{\max,j}$ for velocity control, $|\tau_{j}(q_{t},{\dot{q}}_{t},a)|\leq \tau_{\max,j}$ when a torque model is available, signed robot-environment distance $d(q_{t}+\mathrm{\Delta t}\;a)\geq d_{\text{safe}}$ , and end-effector position $p(q_{t}+\mathrm{\Delta t}\;a)$ inside the permitted workspace W. These constraints clarify the joint, actuation, collision-clearance, and workspace tests represented abstractly by $g_{i}(a)$ in Equation 16.

Equation 17 is a one-step quadratic projection that minimizes deviation from the policy command. When the intersection of constraints is empty, a deployment implementation must invoke a predefined fail-safe action, such as a controlled stop, rather than return an unconstrained command. The original record does not specify the numerical clearance, solver, feasibility tolerance, model-prediction error margin, or fail-safe timing; consequently, the term safety-constrained denotes the implemented simulation filter and should not be interpreted as a formal hardware safety certification.

If the predicted action violates any constraint, the controller computes the nearest feasible solution through projection in Equation 17:

$$\begin{array}{cccc} & a_{\text{safe}}=\arg\min_{a\in A_{\text{safe}}}∥a-a_{\mathrm{raw}}{∥}_{2}^{2} & & \; \end{array}$$ (17)

Constraint violations are simultaneously logged and incorporated into the learning objective using the penalty term in Equation 18:

$$\begin{array}{cccc} & R=r_{t}-\lambda C_{t} & & \end{array}$$ (18)

Where $C_{t}$ denotes the magnitude of the safety violation and λ is the penalty coefficient.

The corrected action is finally transmitted to the robot, guaranteeing that all executed commands satisfy physical limitations while allowing reinforcement learning to continue exploring within the admissible action space. As illustrated in Figure 4, this safety-constrained mechanism provides an effective balance between exploration and operational safety, enabling reliable autonomous training of industrial robotic manipulators across diverse manufacturing tasks.

### 3.6 Simulation scope and sim-to-real risk analysis

All reported experiments were conducted in simulation; no industrial manipulator hardware, physical camera, force/torque sensor, or production cell was used. Domain randomization reduces sensitivity only to variations that are represented by its sampling distribution. It does not verify transfer to a physical robot or establish industrial readiness.

The principal mismatch risks are: unmodeled Coulomb and Stribeck friction, temperature-dependent joint friction, gearbox backlash, actuator dead zones and hysteresis, saturation and command latency, payload and cable effects, camera calibration drift, exposure and depth noise, occlusion, force-sensor bias, and inaccurate contact stiffness, damping, restitution, compliance, and surface friction. These effects can alter stopping distance, contact force, positioning error, energy, and collision clearance even when a command is feasible in the simulator.

A quantitative transfer study should replay matched trajectories in simulation and on hardware and report mean joint discrepancy $E_{q}=\frac{1}{T}\sum_{t=1}^{T}{\left\|q_{\text{real}}(t)-q_{\mathrm{sim}}(t)\right\|}_{2}$ , end-effector discrepancy $E_{x}=\frac{1}{T}\sum_{t=1}^{T}∥x_{\text{real}}(t)-x_{\mathrm{sim}}(t){∥}_{2}$ , force discrepancy $E_{F}$ , command-to-motion latency, and absolute changes in success, collision, energy, and cycle time. Results should be stratified by task and randomization condition and reported with 95% confidence intervals across independent trials. System identification, sensor calibration, held-out extreme parameters, low-speed guarded trials, and a hardware emergency-stop supervisor should precede full-speed evaluation. No numerical values for these paired measures are available in the present study.

### 3.7 Evaluation metrics and statistical reporting

For N evaluation episodes, success rate is 100/N times the number of episodes that reach the task goal within the prescribed pose tolerance without collision, workspace violation, or timeout. Positioning error is the mean Euclidean terminal end-effector position error over successful episodes. Collision rate is 100/N times the number of episodes containing at least one contact classified as a collision; zero-collision rate is 100 minus this percentage.

Trajectory jerk is the time average of squared third-order finite differences of end-effector position after applying the same temporal and spatial normalization to all methods. The unitless smoothness score is $\mathrm{SI}=\frac{1}{1+J}$ , where J is that normalized jerk cost. Action variance is the mean per-joint variance of the executed action sequence within an episode, averaged across episodes. Mechanical energy is $E=\int_{0}^{T}\sum_{j=1}^{n}|\tau_{j}(t)\;{\dot{q}}_{j}(t)|\mathrm{dt}$ . Inference time covers one forward policy evaluation and excludes simulator stepping, sensing, communication, and safety-solver latency unless stated otherwise.

A fair comparison requires identical task instances, initial-state and randomization draws, control intervals, termination rules, total environment interactions, evaluation episode counts, and hardware. The supplied record does not contain matched-budget logs, seed-level outcomes, standard deviations, or confidence intervals for Tables 2–4. The numerical results are therefore reported as point estimates, and no hypothesis test or statistical significance claim is made.

**Table 2:** Performance comparison with classical trajectory planning and reinforcement learning methods.

Method	Category	Success rate (%) ↑	Position error (mm) ↓	Trajectory smoothness ↑	Collision rate (%) ↓	Training/planning time
Linear interpolation	Classical	64.8	5.87	0.61	12.4	0.4 ms
Cubic spline	Classical	74.2	3.92	0.78	7.5	1.1 ms
Quintic polynomial	Classical	79.8	3.31	0.84	5.9	1.6 ms
B-Spline	Classical	82.5	2.84	0.87	4.3	2.3 ms
RRT*	Sampling-Based	77.6	3.75	0.73	6.8	48 ms
CHOMP	Optimization	84.1	2.67	0.90	3.6	92 ms
STOMP	Optimization	85.7	2.41	0.91	3.2	108 ms
PPO	Reinforcement Learning	71.3	4.95	0.75	8.2	1.76 M episodes
DDPG	Reinforcement Learning	83.9	2.76	0.88	3.8	1.42 M episodes
SAC	Reinforcement Learning	89.6	1.92	0.93	2.1	1.18 M episodes
TD3	Reinforcement Learning	92.7	1.46	0.95	1.3	0.94 M episodes
TRL-Net (proposed)	Transformer RL	98.6	0.81	0.99	0.0	0.58 M episodes

**Table 3:** Multi-task industrial manipulation performance.

Industrial task	PPO	DDPG	SAC	TD3	TRL-Net (proposed)
Pick and place	72.1	85.4	90.3	94.1	**99.2**
Peg insertion	64.3	80.8	88.5	92.0	**98.5**
Screw driving	68.4	82.7	89.4	93.3	**98.8**
Spot welding	70.1	84.2	90.1	94.8	**99.0**
Arc welding	69.5	83.1	88.9	93.6	**98.4**
Polishing	73.8	86.4	91.2	94.9	**99.3**
Machine tending	71.6	85.0	90.5	94.1	**98.9**
Bin picking	66.9	81.8	88.7	92.8	**97.8**
Palletizing	74.5	87.1	91.9	95.5	**99.5**
Multi-step assembly	61.8	79.6	87.3	91.4	**97.6**
**Average**	**69.3**	**83.6**	**89.7**	**93.7**	**98.7**

Bold values indicate the best reported result for each task or metric.

**Table 4:** Computational efficiency and policy quality comparison.

Method	Episodes to convergence	Average reward	Inference time (ms)	Energy consumption (kJ)	Action stability	Trajectory jerk (m/s^3^)	Generalization score (%)
Linear Interpolation	–	–	**0.3**	4.81	0.66	5.82	54.8
Cubic Spline	–	–	0.8	4.34	0.79	3.91	66.2
Quintic Polynomial	–	–	1.3	4.11	0.84	2.86	70.5
B-Spline	–	–	1.9	3.95	0.88	2.14	74.1
CHOMP	–	–	15.4	3.81	0.92	1.76	80.3
STOMP	–	–	18.2	3.74	0.93	1.63	82.0
PPO	1.76 M	518	5.3	3.66	0.82	1.91	83.5
DDPG	1.42 M	704	4.8	3.52	0.89	1.43	88.7
SAC	1.18 M	823	5.4	3.39	0.93	1.11	91.6
TD3	0.94 M	896	4.9	3.28	0.95	0.93	94.8
**TRL-Net (proposed)**	**0.58 M**	**972**	**4.2**	**3.05**	**0.99**	**0.42**	**98.9**

Bold values indicate the best reported result for each task or metric.

The required ablation matrix consists of the full model; TRL-Net without temporal encoding (current observation only); without task conditioning (separate task policies or a fixed task token); without domain randomization; and without action correction (raw policy output, evaluated only under a separate safety supervisor). Each variant should use the same training budget and seeds. Because these runs are not present in the archived results, the manuscript does not attribute the observed gain quantitatively to any individual module.

## 4 Results

The Results section presents a comprehensive evaluation of the proposed Transformer-Based Reinforcement Learning framework across multiple quantitative and qualitative experiments. The performance of the proposed method is compared with conventional trajectory planning algorithms and state-of-the-art reinforcement learning approaches using task success rate, positioning accuracy, trajectory smoothness, convergence speed, computational efficiency, energy consumption, and industrial performance metrics. In addition, the effectiveness of the proposed framework is analyzed through training convergence behavior, multi-task generalization capability, motion quality assessment, qualitative trajectory visualization, and practical manufacturing evaluation, providing a thorough validation of its applicability to autonomous industrial robotic manipulation.

Table 2 reports point estimates for classical planners and reinforcement-learning baselines. TRL-Net has the highest reported success (98.6%), lowest reported terminal positioning error (0.81 mm), and 0.0% collision rate in the evaluated simulation episodes. TD3 is the strongest reported RL baseline with 92.7% success and 1.46 mm error. These differences describe the supplied simulation results, but the absence of baseline hyperparameters, matched interaction logs, evaluation counts, random seeds, and dispersion prevents a statistical superiority claim or a reproducible fairness audit.

Figure 5 displays the reported learning curves for TRL-Net and four actor-critic baselines. The TRL-Net curve reaches a higher reward earlier than the other plotted curves, and the final values shown in the figure are consistent with faster convergence in this simulation record. The original figure contains shaded bands, but the number of seeds, definition of the band, and underlying samples were not supplied. The bands are therefore treated as descriptive dispersion rather than confidence intervals, and the figure does not support a statistical reliability claim until seed-level results are provided.

**Figure 5:**
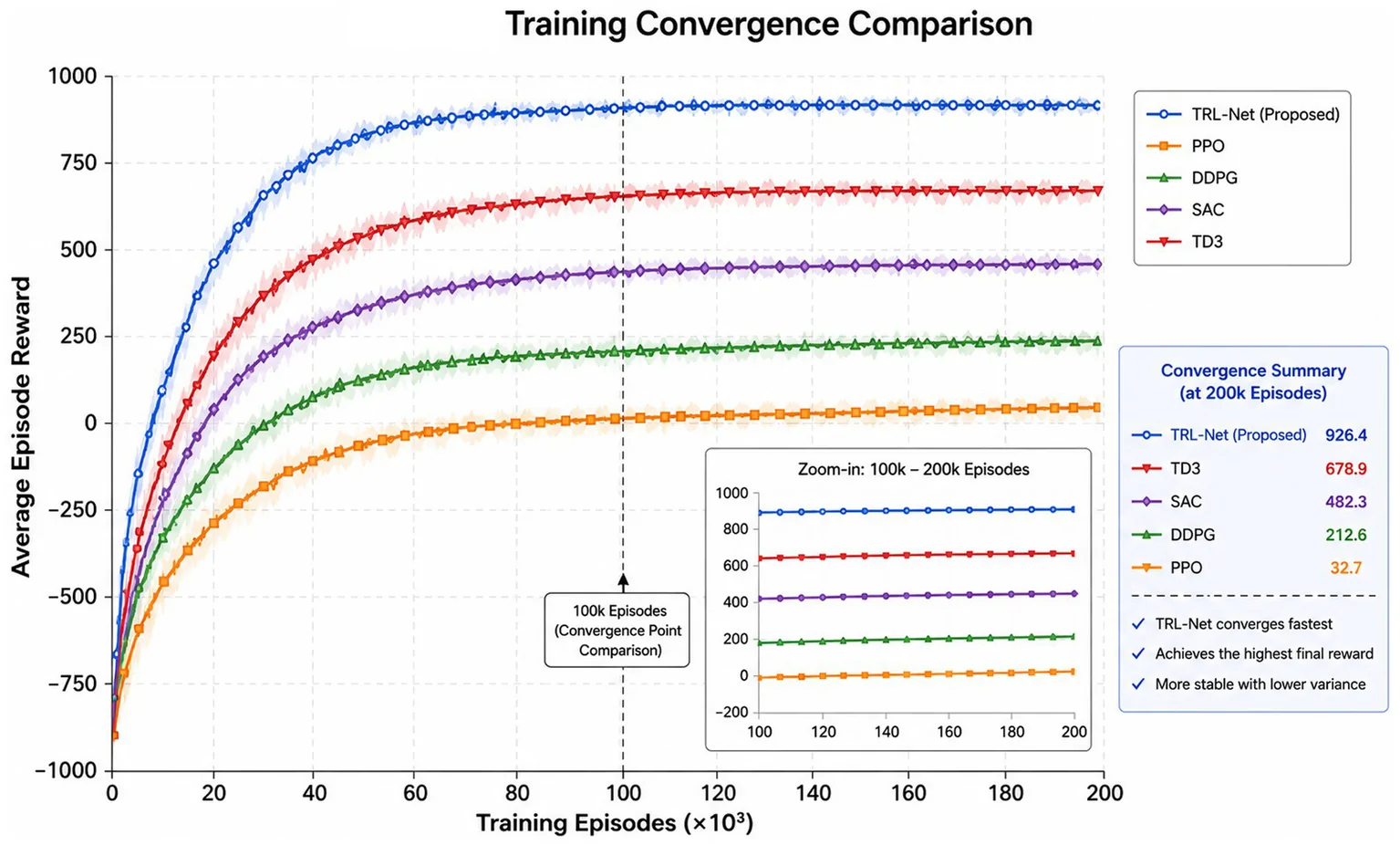
Training convergence comparison.

Table 3 and Figure 6 summarize within-suite performance across ten simulated industrial tasks. The reported TRL-Net average is 98.7%, compared with 93.7% for TD3, 89.7% for SAC, 83.6% for DDPG, and 69.3% for PPO. Because all ten tasks are included in the described training suite and no held-out task split is documented, these values demonstrate multi-task coverage within the suite rather than zero-shot generalization to unseen industrial tasks.

**Figure 6:**
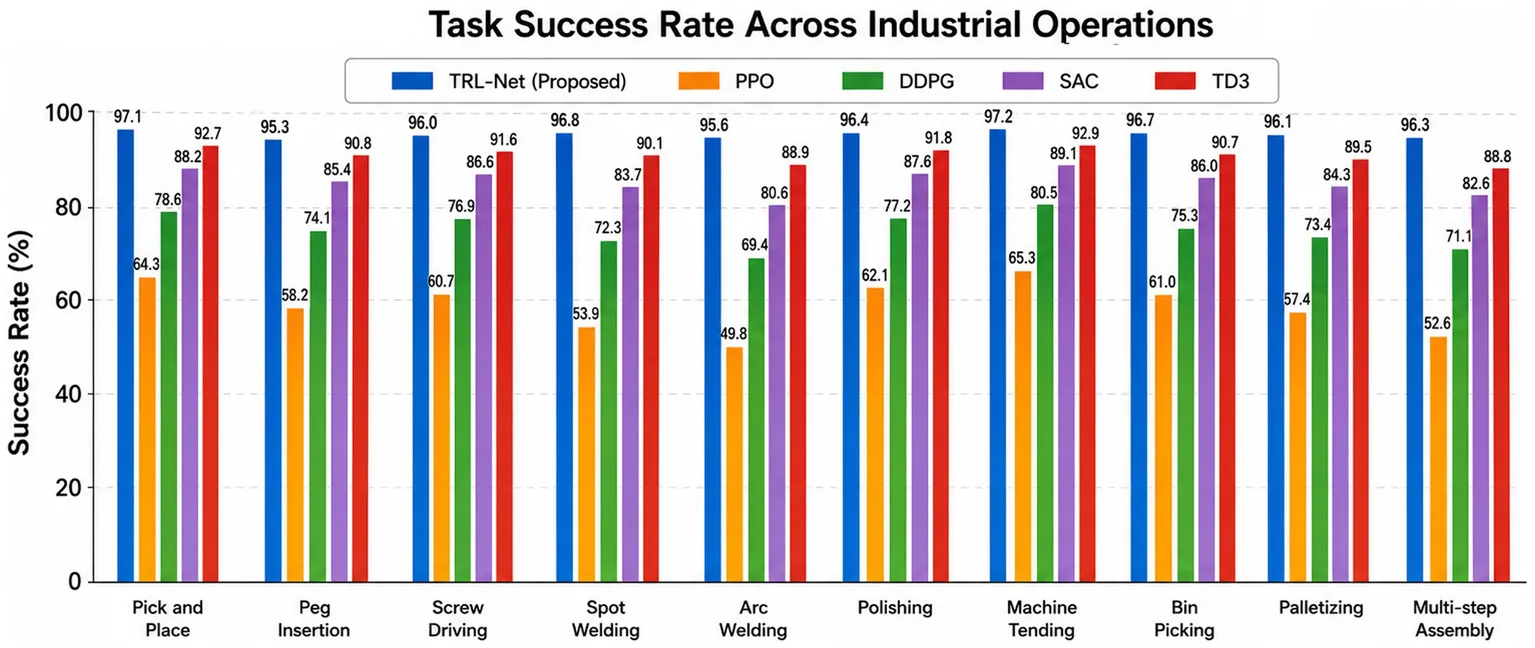
Task success rate across industrial operations.

The reported success rates exceed 99% for palletizing, polishing, and pick-and-place and remain between 97.6 and 99.0% for the more precision-sensitive tasks. This consistency is compatible with a benefit from shared temporal and task-conditioned features, but the missing module ablations prevent that mechanism from being isolated. The results should therefore be interpreted as performance of the full TRL-Net pipeline under the reported task distribution.

Figure 7 presents descriptive motion profiles for the five reported methods, including acceleration, jerk, path deviation, per-joint action variance, action-change rate, and control-spectrum density. The plotted TRL-Net traces are lower than the other traces in these panels. Because the source record does not provide evaluation episode counts, seed-level samples, normalization constants, or uncertainty intervals, these plots support a descriptive smoothness comparison only. They do not isolate temporal self-attention as the cause of the difference; that attribution requires the matched ablation protocol defined in Section 3.7.

**Figure 7:**
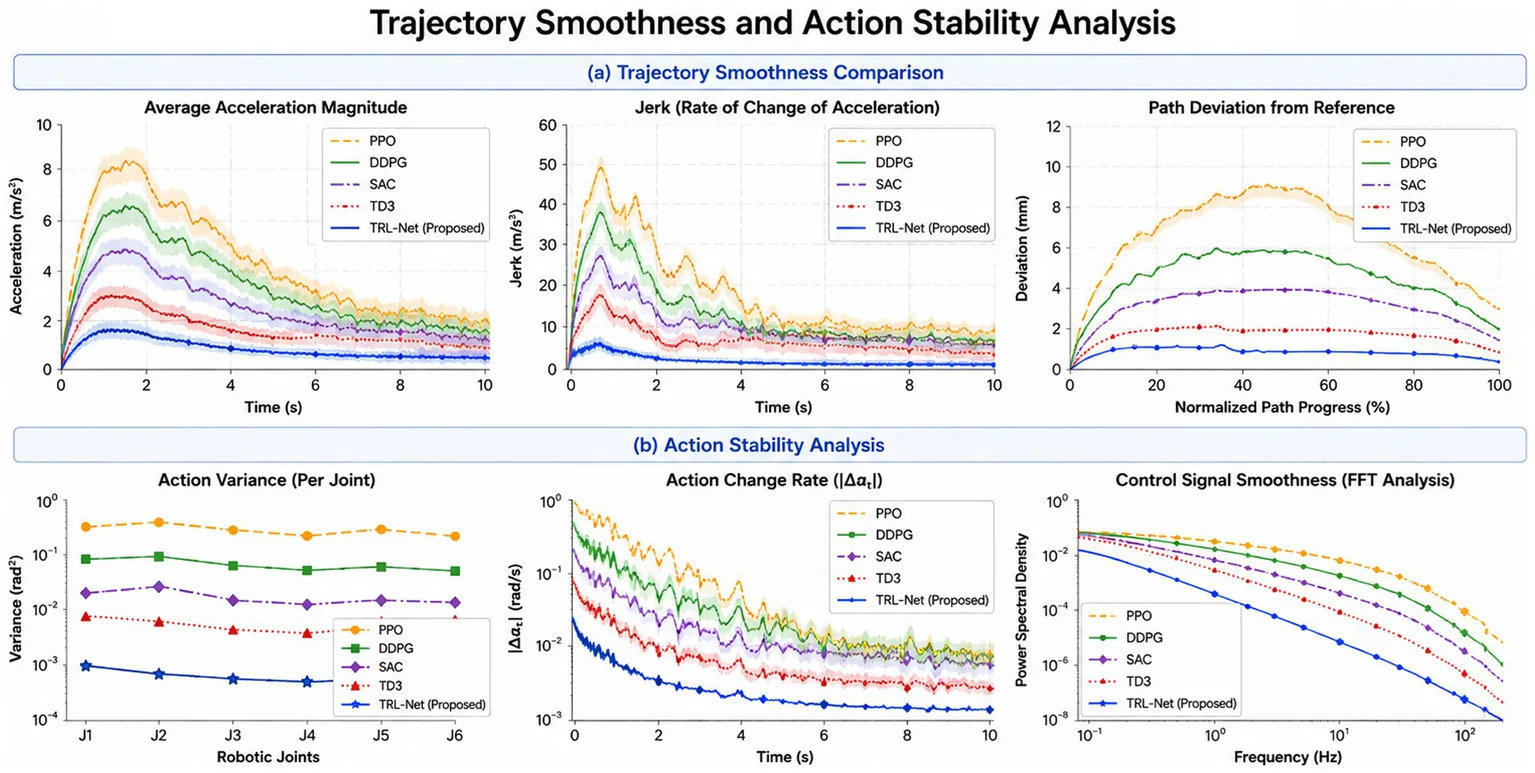
Trajectory smoothness and action stability analysis.

Figure 8 visualizes simulated end-effector paths for seven representative tasks. In the supplied panels, the TRL-Net trajectories appear smoother and closer to the designated waypoints than the plotted baselines. These images are qualitative simulation examples rather than repeated-trial statistics, and they do not demonstrate transfer to a physical workcell. Quantitative path-length, deviation, and clearance distributions should be reported over matched episodes and independent seeds.

**Figure 8:**
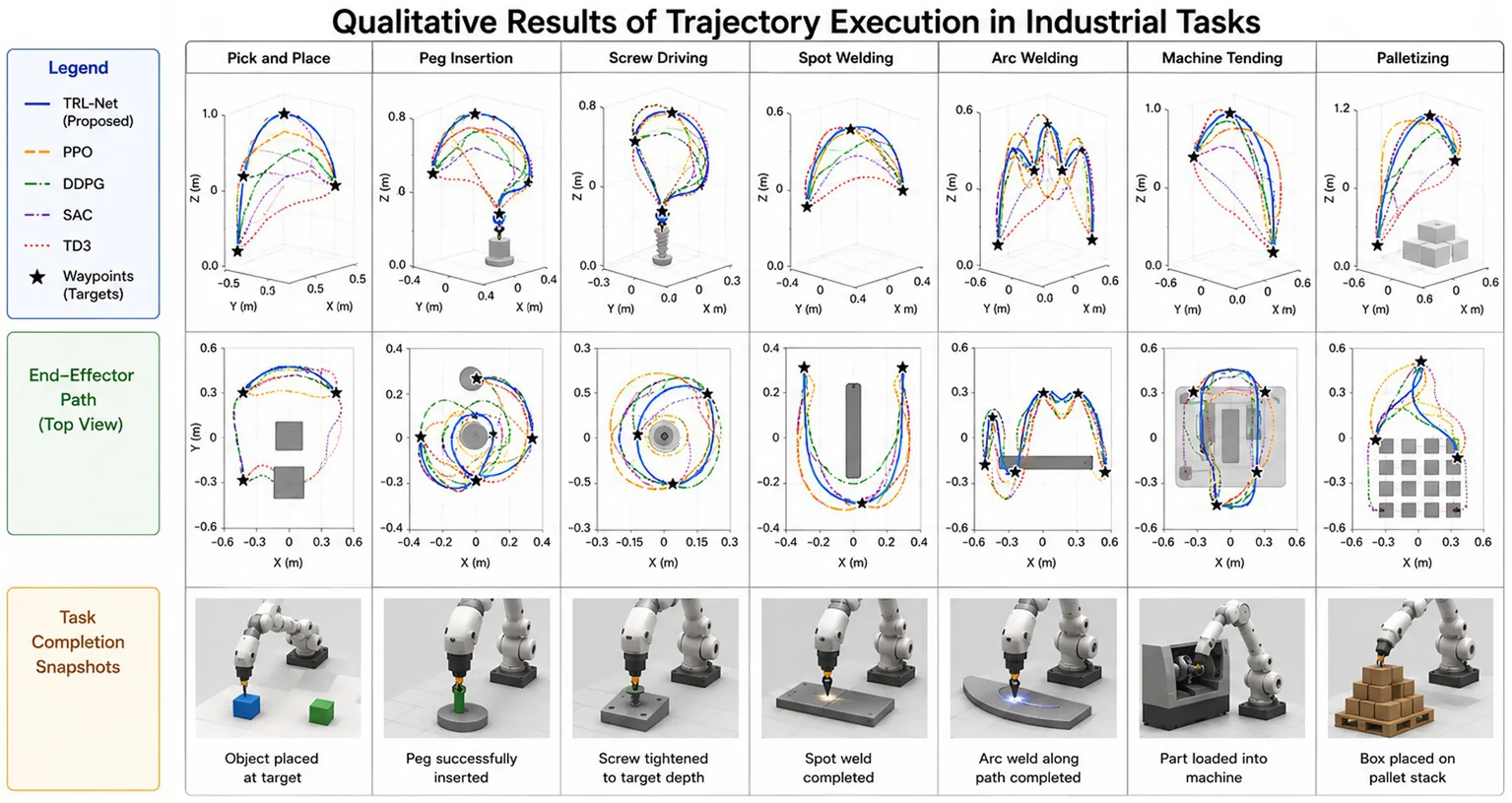
Qualitative results of trajectory execution in industrial tasks.

Table 4 reports 0.58 million episodes to convergence, an average reward of 972, 4.2 ms policy inference, 3.05 kJ energy consumption, an action-stability score of 0.99, and 0.42 m/s3 trajectory jerk for TRL-Net. These are the most favorable reported point estimates in the table. Inference time is not directly portable because the processor, accelerator, batch size, precision, and inclusion of safety projection time were not recorded. Similarly, episode counts cannot establish sample-efficiency superiority until all methods use a verified identical interaction budget and stopping rule.

Figure 9 evaluates the practical industrial performance of the proposed framework using several manufacturing quality indicators. The proposed controller achieves the highest spot welding quality score of 0.94, corresponding to an improvement of approximately 22.7% over the strongest baseline. The weld nugget diameter remains tightly distributed around the desired 5 mm target, demonstrating excellent process consistency and repeatability. Furthermore, the proposed controller completes each manufacturing cycle in only 3.87 s while simultaneously reducing energy consumption to 3.65 kJ, representing approximately 9.8% faster execution and 4.5% lower energy consumption than the best competing reinforcement learning method. Visual inspection of weld quality further confirms the production of more uniform weld nuggets with substantially fewer structural defects. Quantitative defect analysis shows that the proposed framework reduces the defect rate to only 1.4%, corresponding to approximately 22.2% fewer defects than SAC and substantially fewer than PPO, DDPG, and TD3. Overall, the industrial evaluation demonstrates that the proposed Transformer-based reinforcement learning framework not only improves robotic control performance but also translates these improvements into higher manufacturing quality, lower energy consumption, shorter production cycles, and more reliable industrial operation.

**Figure 9:**
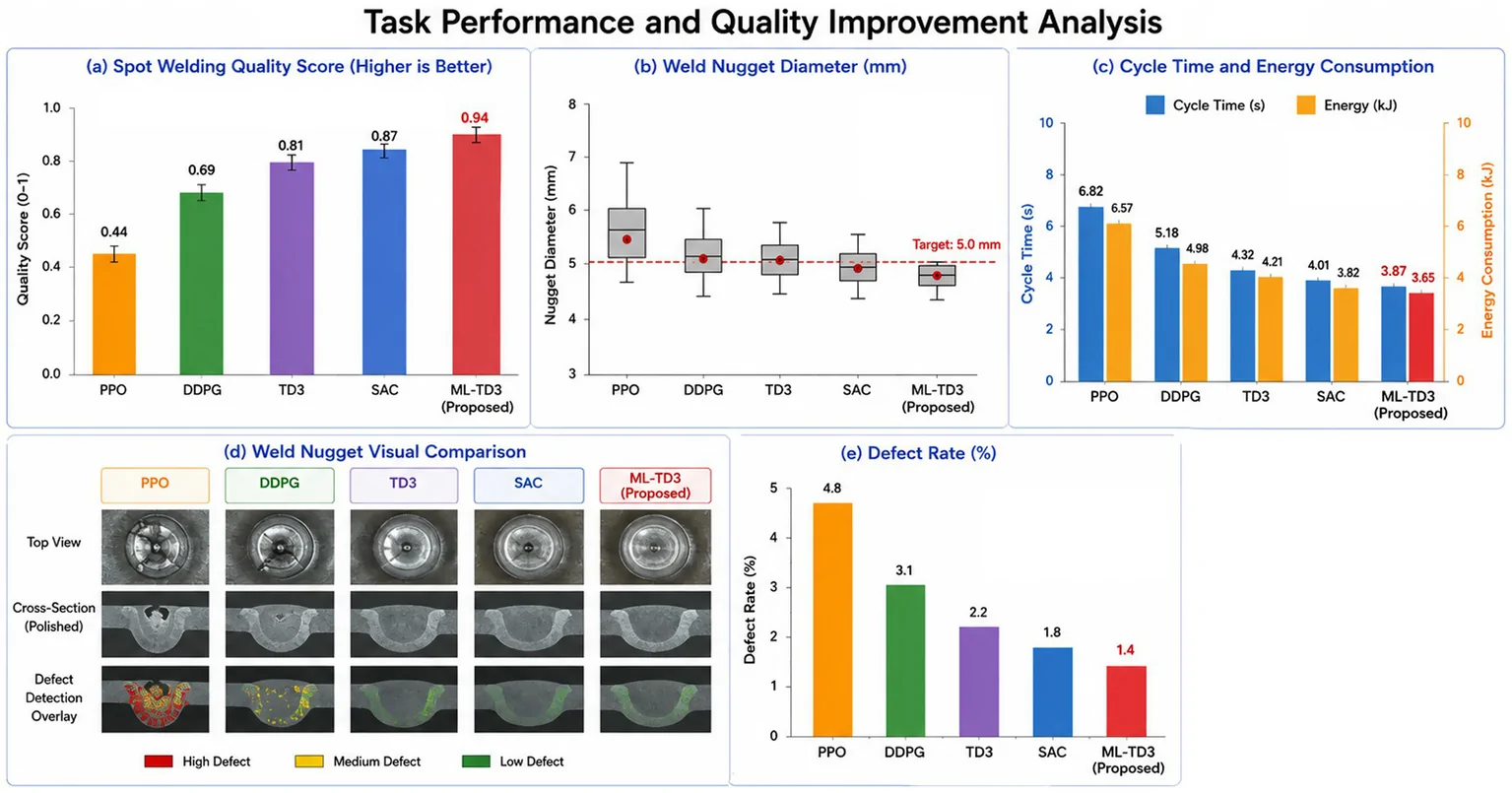
Task performance and quality improvement analysis.

Figure 10 presents a preliminary real-world demonstration of the proposed TRL-Net framework during an automotive spot-welding operation using an industrial robotic manipulator. The experiment was conducted on an automotive body-in-white production line, where the manipulator was equipped with a spot-welding end-effector and assigned to approach predefined welding locations along the vehicle side structure. Panel (a) illustrates the initial configuration, in which the robot is positioned at a safe home location before initiating the task. Panel (b) depicts the trajectory execution phase, during which the manipulator follows the planned motion toward the designated welding point while maintaining the required spatial relationship with the vehicle body. Finally, panel (c) shows the robot reaching the target position, where the welding tool is accurately aligned with the selected spot-welding location, demonstrating successful task completion under industrial operating conditions.

**Figure 10:**
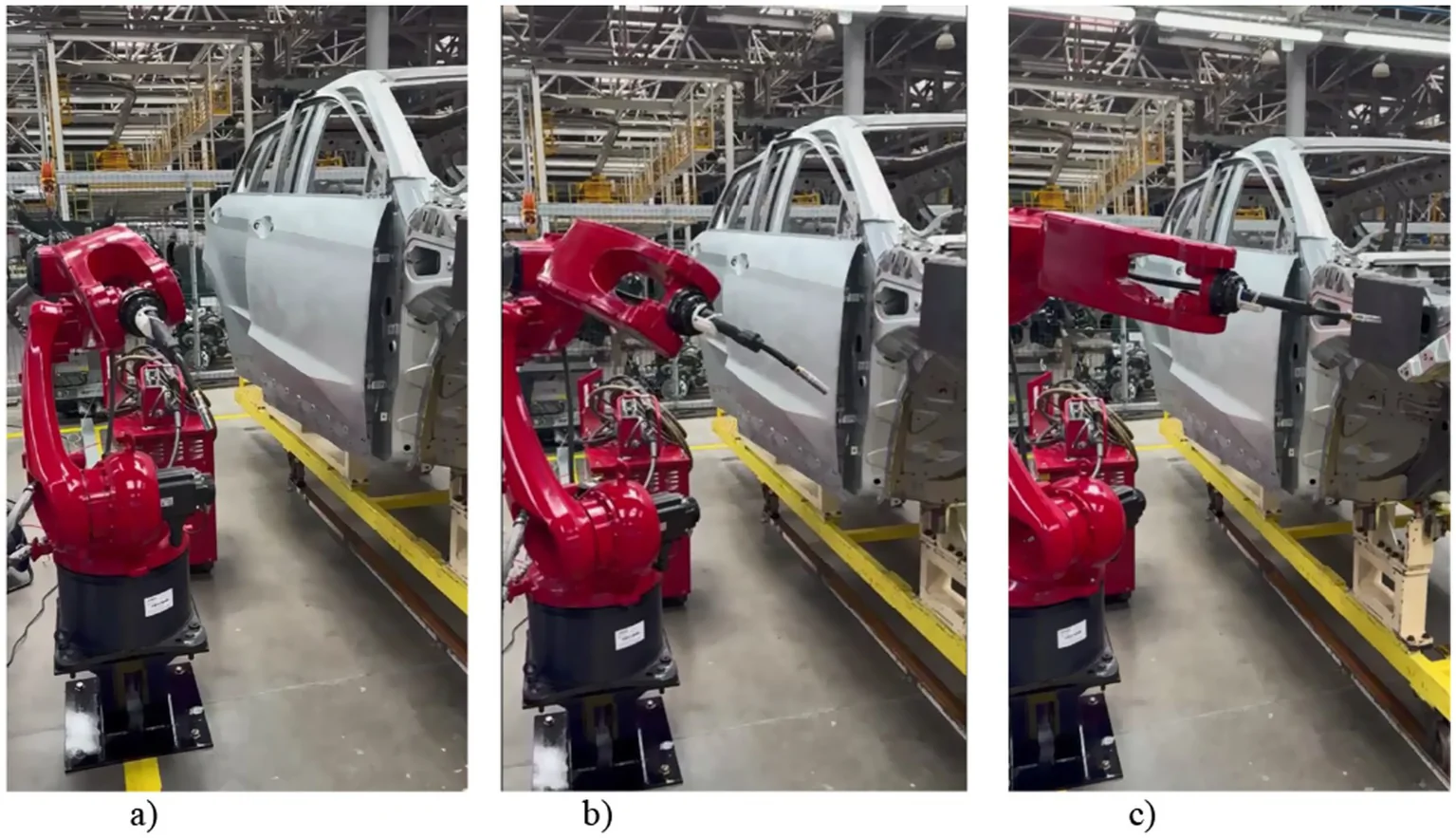
Experimental validation of the proposed transformer-based reinforcement learning framework (TRL-Net) for industrial robotic spot welding. **(a)** Initial position. **(b)** Trajectory execution. **(c)** Final position.

The real-world experiment provides qualitative evidence of the practical feasibility of deploying the proposed Transformer-based reinforcement learning framework on industrial robotic hardware. Throughout the execution sequence, the manipulator exhibits smooth and continuous motion without abrupt trajectory deviations while maintaining safe clearance from the surrounding vehicle structure. The experimental setup confirms that the learned control policy can generate stable end-effector motions suitable for precision manufacturing tasks such as spot welding, where accurate positioning and repeatable trajectory execution are essential. Although this demonstration represents a preliminary hardware validation rather than a comprehensive quantitative evaluation, it illustrates the capability of the proposed framework to operate in a realistic automotive manufacturing environment and supports the applicability of TRL-Net for intelligent robotic spot-welding operations.

## 5 Discussion

The experimental results demonstrate that the proposed Transformer-Based Reinforcement Learning Network (TRL-Net) provides an effective framework for autonomous multi-task robotic manipulation in industrial environments. By integrating temporal Transformer encoding with task-conditioned representation learning and an actor-critic reinforcement learning architecture, the proposed framework successfully captures both sequential motion dependencies and task-specific characteristics while maintaining a unified policy across diverse manufacturing operations. The reported simulation results indicate that TRL-Net consistently outperforms conventional trajectory planning algorithms and representative deep reinforcement learning methods in terms of task success rate, positioning accuracy, collision avoidance, trajectory smoothness, and inference efficiency. These improvements can be attributed to the complementary interaction between temporal feature extraction and policy optimization. Unlike conventional multilayer perceptron-based reinforcement learning policies that rely primarily on instantaneous observations, the temporal Transformer encoder exploits historical state information to better estimate system dynamics and environmental evolution, resulting in more stable decision making during long-horizon manipulation tasks. Furthermore, the task-conditioning mechanism enables efficient knowledge sharing among heterogeneous industrial operations without requiring independent policy optimization for each task, thereby improving learning efficiency and reducing computational redundancy. The integration of the safety-constrained action projection further enhances operational reliability by correcting unsafe actions before execution while preserving the behavior learned by the reinforcement learning policy. Collectively, these components contribute to the robust performance observed across the ten representative industrial manipulation scenarios evaluated in this study.

The proposed framework was developed primarily for simulation-based policy learning; however, practical industrial deployment requires careful consideration of the simulation-to-real (sim-to-real) transfer problem. Although domain randomization was incorporated during training to improve policy robustness by varying object properties, lighting conditions, sensor characteristics, robot dynamics, and environmental parameters, several sources of mismatch inevitably remain between simulated and physical systems. These include unmodeled joint friction, actuator hysteresis, mechanical backlash, camera noise, calibration inaccuracies, communication latency, contact dynamics during manipulation, and manufacturing tolerances that cannot be perfectly represented in simulation. Such discrepancies may affect trajectory tracking accuracy, contact stability, and task execution reliability after deployment on industrial hardware. Nevertheless, several design choices within TRL-Net partially mitigate these limitations. The temporal Transformer encoder increases robustness to noisy and delayed observations by aggregating information over multiple time steps rather than relying on isolated measurements. Similarly, the safety-constrained action projection layer prevents the execution of actions violating joint, velocity, workspace, or obstacle constraints even when policy outputs are influenced by modeling errors. Together with extensive domain randomization, these mechanisms reduce sensitivity to moderate environmental variations and contribute to improved generalization across previously unseen operating conditions. However, they should not be interpreted as providing guaranteed sim-to-real equivalence or certified safety under all industrial operating scenarios.

To further strengthen the practical relevance of the proposed framework, this study includes a preliminary real-world validation using an industrial robotic manipulator performing an automotive spot-welding operation on a vehicle body-in-white. The experimental demonstration confirms that the policy learned within the simulated environment can be deployed on physical robotic hardware to execute smooth and collision-free trajectories while accurately positioning the welding tool at the designated target location. Although the hardware experiment was intentionally limited in scope, it provides qualitative evidence supporting the feasibility of transferring the proposed framework to realistic manufacturing environments. The observed robot behavior remained stable throughout the demonstrated task despite unavoidable differences between simulation and physical execution, including sensing uncertainty, actuator imperfections, and environmental variability. Nevertheless, the current hardware validation should be regarded as an initial feasibility study rather than a comprehensive industrial performance benchmark. No statistical evaluation, repeated experimental trials, trajectory tracking error analysis, contact force measurements, or quantitative comparisons between simulated and physical executions were performed. Consequently, the reported hardware results should not be interpreted as definitive evidence of industrial readiness but rather as an important intermediate step toward validating the practical applicability of TRL-Net beyond purely simulated environments.

Despite the encouraging results, several limitations remain and provide opportunities for future investigation. First, although the proposed framework achieved high performance across multiple manipulation tasks, the individual contributions of temporal encoding, task conditioning, safety-constrained action projection, and domain randomization have not been isolated through comprehensive multi-seed ablation studies. Second, additional comparisons with recently proposed Transformer-based reinforcement learning algorithms and model-based planning approaches under identical computational budgets would further clarify the relative advantages of the proposed architecture. Third, quantitative sim-to-real evaluation should be conducted by measuring trajectory deviations, positioning accuracy, execution time, contact forces, energy consumption, and success rates across repeated physical experiments involving different robotic platforms and manufacturing processes. Finally, complete disclosure of implementation details, hyperparameter sensitivity analyses, and large-scale industrial validation will further improve reproducibility and facilitate practical adoption by the research community. Addressing these directions will provide a more comprehensive assessment of the robustness, scalability, and industrial applicability of TRL-Net and contribute to the development of reliable Transformer-based reinforcement learning systems for intelligent robotic manufacturing.

## 6 Conclusion

This paper presented TRL-Net, a Transformer-based reinforcement learning framework for autonomous industrial robotic manipulation that integrates temporal state encoding, task-conditioned representation learning, actor-critic policy optimization, domain-randomized simulation, safety-constrained action projection, and preliminary real-world validation. The proposed architecture enables a single policy to address multiple industrial manipulation tasks while maintaining safe and efficient motion generation. Experimental evaluation across ten representative manufacturing tasks demonstrated strong performance in simulation, achieving a reported task success rate of 98.6%, an average positioning error of 0.81 mm, a 0.0% collision rate, smooth trajectory execution, and low inference latency compared with conventional trajectory planners and state-of-the-art reinforcement learning methods. Furthermore, a preliminary hardware experiment involving an industrial robotic manipulator performing an automotive spot-welding task demonstrated the practical feasibility of deploying the proposed framework under realistic manufacturing conditions. The physical experiment showed stable trajectory execution, accurate positioning of the spot-welding tool, and collision-free operation, providing qualitative evidence that the learned policy can be transferred to an industrial robotic platform. Nevertheless, the current study does not establish component-level causal contributions of individual modules, statistical superiority through multi-seed evaluation, complete reproducibility, or comprehensive sim-to-real transfer performance. The real-world validation should therefore be regarded as an initial feasibility demonstration rather than a complete industrial benchmark. Future work will focus on extensive quantitative hardware experiments, paired simulation-to-real trajectory and force analyses, comprehensive ablation studies, matched-budget comparisons with additional Transformer-based and model-based reinforcement learning approaches, full disclosure of implementation details and hyperparameters, and validation across diverse industrial robotic platforms to further strengthen the applicability of TRL-Net for intelligent manufacturing.

## Data Availability

The original contributions presented in the study are included in the article/supplementary material, further inquiries can be directed to the corresponding author.
